# Anion Exchange Membrane Water Electrolysis Using a Catalyst-Coated Membrane Cathode

**DOI:** 10.1021/acselectrochem.5c00280

**Published:** 2025-09-25

**Authors:** Habin Park, Shane Harris, Paul A. Kohl

**Affiliations:** 1372 Georgia Institute of Technology, School of Chemical and Biomolecular Engineering, Atlanta, Georgia 30332-0100, United States

**Keywords:** Water electrolysis, anion-exchange membrane, catalyst-coated membrane, cross-linking, durability

## Abstract

A catalyst-coated membrane (CCM) approach to electrode fabrication for high pH water electrolysis offers enhanced interfacial contact between the catalyst layer and the membrane surface in comparison to the catalyst-coated substrate (CCS) electrode configuration. The CCM facilitates enhanced ionic and water transport between the cathode and the anion exchange membrane (AEM). This advantage is particularly significant with AEM water electrolysis (compared to proton exchange membrane water electrolysis) because the cathode typically operates under dry conditions and relies solely on diffusive water transport across the AEM from the liquid-fed anode. This study presents a direct performance comparison between CCS and CCM cathode configurations using identical hydrogen evolution reaction (HER) catalysts and other components. The use of a pseudo-reference electrode integrated into the membrane electrode assembly enabled detailed analysis of the CCM cathode polarization behavior. Surface characterization provided insight into the degradation mechanisms associated with the CCM configuration. Optimization of the cathode ionomer cross-link density improved both the cathode polarization performance and the electrolysis device durability. Further optimization of the HER catalyst loading in the CCM cathode resulted in additional gains in the electrolysis efficiency. Collectively, these findings offer valuable guidance for the design and fabrication of high-performance, durable AEM electrolysis CCMs.

## Graphical abstract

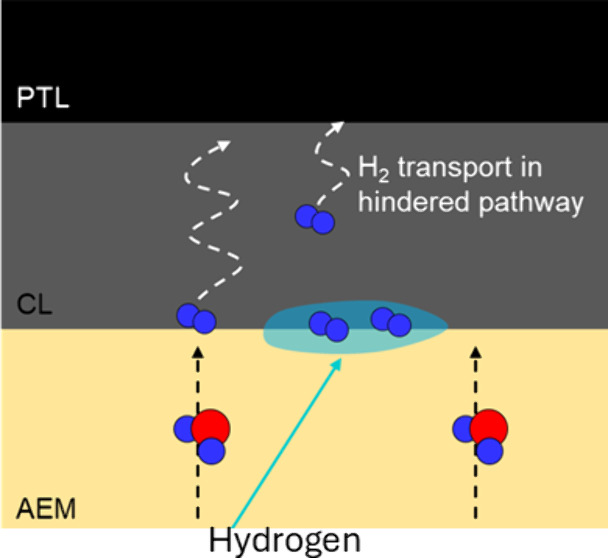

## Introduction

Green hydrogen is emerging as a pivotal energy carrier in the transition toward net-zero carbon emissions by 2050, offering a carbon-free pathway for energy storage, chemical synthesis, and transportation. Among the various production methods, water electrolysis powered by renewable energy is considered an economical and sustainable approach for green hydrogen generation.^1–3^

Anion exchange membrane water electrolysis (AEMWE) combines the advantages of both conventional alkaline and (solid-polymer) proton electrolyte membrane (PEM) electrolysis. AEMWE enables the use of non-platinum group metal catalysts for the oxygen evolution reaction (OER) due to the high pH environment and supports high current density operation (>1 A/cm^2^) at modest temperature, similar to PEM electrolysis using a zero-gap membrane electrode assembly (MEA) design.^4^ Unlike PEM electrolysis, which relies on perfluorinated polymer electrolytes (e.g., Nafion), AEMWE uses hydrocarbon-based anion-conducting polymers, making it more cost-effective and environmentally friendly.^5^

Recent AEMWE advancements have demonstrated operation below 2.0 V at >2 A cm^–2^ current density with good long-term durability at >1 A cm^–2^.^6–9^ Achieving stable performance using a dry cathode to produce pressurized hydrogen production requires high water transport to the cathode and stable electrodes. Our previous findings indicate that a dry cathode can suffer from water transport and ionic conductivity limitations because the conduction of water and hydroxide ions relies on the ionomer within the cathode. The introduction of non-electroactive salts in the anolyte was shown to improve water transport toward the cathode and reduce the cell overpotential.^8,10^

High-current-density AEMWE performance is typically constrained by Ohmic resistance and mass transport limitations.^11^ Thus, enhanced ionic conductivity and mass transport within the cathode are critical for improving AEMWE efficiency and durability. In AEMWE, hydroxide ions migrate from the hydrogen-evolving cathode to the oxygen-evolving anode, while water diffuses from the water fed anode to the dry cathode. Strategies to enhance ion transport include increasing the ion exchange capacity (IEC) of ionomers,^12,13^ optimizing anolyte salt concentration,^10^ controlling catalyst layer thickness,^14^ and employing a catalyst-coated membrane (CCM) configuration.^15,16^ The CCM approach adapted from PEM fuel cell and electrolysis systems involves direct deposition of the catalyst layer onto the membrane. This improves catalyst utilization, reduces interfacial resistance, and enhances mass transport.^15,16^ The CCM electrode also potentially improves the integration of the catalyst with the membrane, compared to catalyst-coated substrate (CCS) methods, because roll-to-roll manufacturing methods can more easily be used.^17^ CCM fabrication methods include the decal-transfer method, which avoids membrane swelling, and the direct spray coating method, which offers a scalable alternative with catalyst layer porosity.^17,18^

In this study, the CCM cathode performance enhancement was studied using a pseudo-reference electrode (pRE) integrated into the electrolysis cell. A CCS oxygen-evolving anode was used because it is less affected by water and ion transport limitations compared to the cathode due to the presence of a liquid anolyte. Performance and durability of the CCM cathode were benchmarked against our previously developed CCS cathode, which exhibited excellent durability and low-voltage operation at 1 A cm^–2^, even in lower pH alkaline electrolytes.^8^ While both configurations use a self-adhesive ionomer, covalent bonding between the cathode catalyst and the porous transport layer (PTL) substrate occurred only in the CCS cathode, leading to differences in mechanical durability. The mechanical degradation mechanisms of the CCM cathodes were investigated. It was shown that cross-linking within the cathode ionomer improved the performance and durability at high current density, such as 1.5 A cm^–2^. The impact of catalyst loading density on electrolysis performance and durability was also assessed. This work offers a comprehensive comparative analysis of CCM and CCS cathode architectures, providing insights into scalable, durable, and energy-efficient cathode designs for reliable green hydrogen production.

## Experimental Section

Anion-conducting poly­(norbornene) ionomers were synthesized by following the method reported by Mandal et al.^19^ The ester functional group in the ionomer was converted to a pendant carboxylic acid via the reaction with hydrochloric acid. Subsequent esterification with bisphenol-A diglycidyl ether (BPADGE) produced the cross-linked ionomer. The final ionomer, designated as hydrogen evolution reaction (HER)-A, consisted of a monomer composition ratio of 20:60:20 for butyl norbornene (BuNB), bromobutyl norbornene (BrBuNB), and carboxylic acid norbornene (CANB), respectively. HER-A was used as the ionomer component in the hydrogen evolution reaction (HER) cathodes. The ion exchange capacity (IEC), number-average molecular weight (Mn), and polydispersity index (*Đ*) were characterized via nuclear magnetic resonance (NMR) spectroscopy.^12^

The oxygen evolving anodes were fabricated using the solvent-cast method as previously described.^12^ The anodes were prepared with 0.5 mg cm^–2^ NiFe_2_O_4_ catalyst (Sainergy) and 0.61 mg cm^–2^ BPADGE adhesive. Electrode inks were sonicated for 1 h in an ice bath before spray-coating onto a nickel felt (Technetics Group). The anodes were thermally cured in an oven at 160 °C for 1 h to promote esterification between BPADGE and CANB.

The HER cathodes were fabricated using the solvent-cast method as previously described.^12^ The cathodes had 1.2 mg cm^–2^ of Pt_3_Ni catalyst (40 wt % Pt on carbon, Sainergy), 0.6 mg cm^–2^ HER-A ionomer, and 0.25 mg cm^–2^ BPADGE adhesive. Various mol %’s of tetramethyl hexanediamine (TMHD) cross-linker were added to the electrode ink to cross-link the ionomer within the electrode. CCS cathodes were made by spraying the cathode ink onto non-wetproofed carbon paper (AvCarb MGL280, Fuel Cell Store). A mechanical jig made of stainless-steel plates with a window was used to hold the anion exchange membrane (AEM) for the spray-coating. The fabricated CCS and CCM cathodes were thermally cured in an oven at 160 and 85 °C for 1 and 48 h, respectively. The CCS and CCM cathodes were aminated in 45 wt % TMA solution for 24 h before use.

SEM imaging of the HER cathodes was performed using a Phenom XL G2 instrument (Thermo Fisher Scientific) operated at 15 kV. Samples were neither aminated nor ion-exchanged prior to analysis. Prior to imaging, electrodes were vacuum-dried at 60 °C overnight. Cross-sectional SEM images were obtained by cutting samples with a scalpel. The void volume ratio of the catalyst layers in CCM vs CCS electrodes was estimated using the following eq 1.

1 $$\frac{V_{void,CCM}}{V_{void,CCS}}=\frac{T_{CCM}-\frac{\rho_{CL}}{D_{CL}}}{T_{CCS}-\frac{\rho_{CL}}{D_{CL}}}$$

In eq 1, *V*_void_ is the pore volume (cm^3^), *T* is the thickness of catalyst layer (cm), ρ_CL_ is the loading density of the catalyst (mg/cm^2^), and *D*_CL_ is the density of the catalyst layer composed of catalyst and polymers (mg/cm^3^). Energy dispersive spectroscopy (EDS) was used to analyze elements incorporated in electrodes.

The membrane electrode assembly (MEA) electrode area was 4 cm^2^. Both CCS and CCM electrodes, as well as the PNB^(R)^-R45 anion exchange membranes (thickness ≈ 45 μm, IEC = 3.5 mequiv/g), were ion exchanged in 1.0 M KOH prior to MEA assembly. The MEAs were assembled between gold-coated stainless steel flow fields and secured using bolts torqued to 1.5 ft-lb. A 250 μm diameter platinum wire (Alfa Aesar) was integrated into the cathode with Tefzel gaskets to serve as a pseudo-reference electrode (pRE). The wire contacted the membrane on the cathode side and was positioned 2 mm from the cathode PTL.

Electrochemical measurements were performed by using a DC power supply (N5742A, Keysight). A DC current was applied, and the resulting cell voltage was recorded. Polarization curves were obtained by applying a current for 1 min, followed by stepping to a higher current value. Prior to testing, cells were conditioned at 0.1 A cm^–2^ for 30 min. Anode and cathode potentials versus the pRE were measured under open-circuit conditions using a potentiostat (SP300, Bio-Logic). The voltage of each cell was recorded at 1.5 A cm^–2^. The uncertainty in the voltage measurement was +/–10 mV. Multiple cells of critical configurations were tested to ensure the results are reproducible. The reproducibility of the voltage at the critical current of 1.5 A cm^–2^ was estimated to be +/–1%. Two electrode (anode vs cathode) electrochemical impedance spectroscopy (EIS) was conducted at constant current, 1.5 A/cm^2^, because the transport limitations can only be observed at high current density. The data is presented in Nyquist plots. The double layer capacitance (*C*_dl_, F cm^–2^) was calculated from the maximum in the out-of-phase impedance value, eq 2.

2 $$C_{dl}=\frac{1}{2\pi f_{peak}R_{\mathrm{int}}}$$

In eq 2, *f*_peak_ is the peak frequency (Hz) of out-of-phase semicircles of Nyquist plots and *R*_int_ is the interfacial resistance (Ω cm^2^), which is the diameter of out-of-phase semicircles.

## Results and Discussion

The electrochemical performances of the CCS and CCM cathodes were evaluated holding all other materials and conditions the same, including the OER cathode, anolyte, and AEM. Figure 1 compares the electrolysis performance of the CCS and CCM cathodes. The polarization curves in Figure 1a show two distinct regions: the activation region (<0.15 A cm^–2^) and the ohmic/mass transport region (>0.3 A cm^–2^). While the CCM cathode exhibited a slightly higher activation overpotential at low current compared to the CCS cathode, it shows lower polarization loss, as seen by the smaller slope in the ohmic and mass transport regions. The smaller slope at high current indicates improved water and ionic transport between the cathode and AEM due to the modified morphology of the CCM catalyst layer, which is deposited directly onto the AEM surface. The anode and cathode potentials were each recorded by use of the reference electrode, Figure 1b. The anode potentials, top two curves in Figure 1b, are nearly identical for the two cells, while the cathode potential, bottom two curves in Figure 1b, shows a different slope for the CCM and CCS electrodes. The improvement in cell voltage observed in Figure 1a for the CCM electrode is clearly due to lower losses at the CCM cathode compared with the CCS cathode. The Nyquist plots in Figure 1c corroborate this finding, showing that the CCM cathode has significantly lower high-frequency resistance (HFR) compared to that of the cell with the CCS cathode. The HFR is the *x*-intercept on the left side of the impedance loop and corresponds to the resistance in series with parallel resistance-capacitance circuit elements. The difference between the high frequency *x*-intercept (left side *x*-intercept) and low frequency *x*-intercept (right side *x*-intercept) captures the in-phase impedance for the parallel resistance-capacitance circuit elements, which includes the charge transfer resistance. At a current density of 1.5 A cm^–2^, the voltage difference between the CCM and CCS cells (Δ*E*_cell_) was 102 mV, as shown in Figure 1a. The EIS, Figure 1c, shows a 128 mV improvement in ohmic losses (Δη_e_) for the CCM cell, compared to the CCS cell, due to an improvement in the contact between the AEM and the cathode. The remaining voltage loss, −26 mV, is attributed to a change in the interfacial overpotential (Δη_Int_). This is consistent with previous findings by de Pablo et al., which described the role of water networks in enhancing ion conductivity in anion-conducting polymer electrolytes.^20^

**Figure 1:**
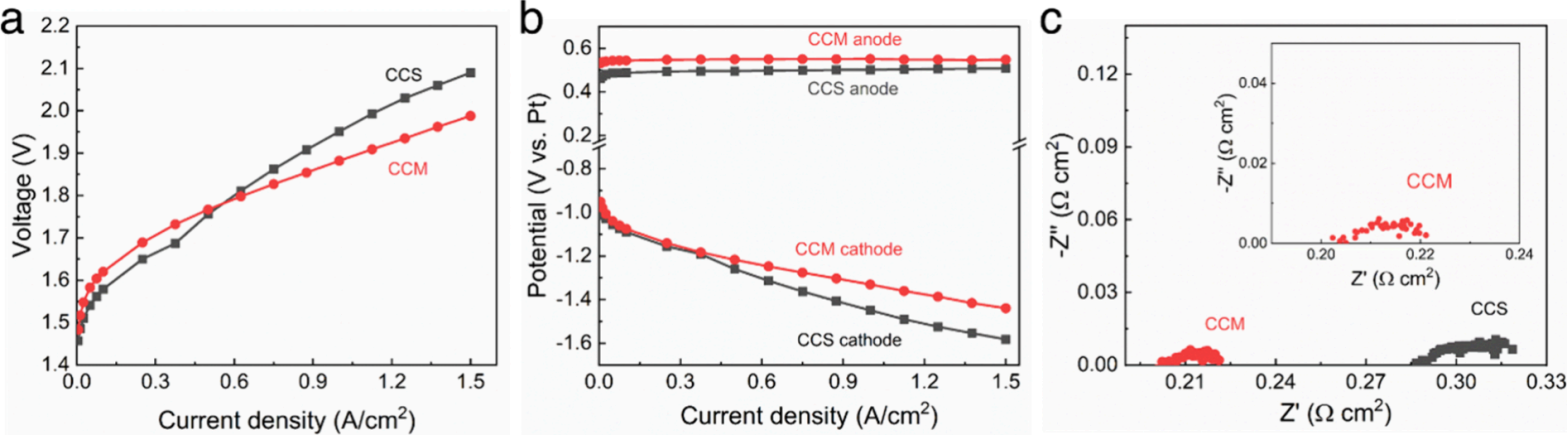
Water electrolysis performance comparison between CCS and CCM. Cells were tested at 60 °C with a dry cathode: (a) polarization curves, (b) anode (top) and cathode (bottom) polarization curves measured with Pt pRE, and (c) EIS at 1.5 A cm^–2^ with expanded view of CCM in the inset.

The morphology of the CCM and CCS electrodes was examined. Figure 2 shows SEM images of the CCS and CCM cathodes. The CCS catalyst layer had large pores (>50 μm), while the CCM structure had micro-cracks (∼1 μm), consistent with deposition on the smooth AEM surface (Figure 2a,b). The cross-sectional image of the CCM electrode, Figure 2c, shows a dense, 25 to 30 μm CCM catalyst layer in contact with the membrane compared to the ca. 100 μm thick, higher porosity CCS layer.^12^ The estimated pore volume in the CCM electrode was less than 30% of that in the CCS electrode.

**Figure 2:**
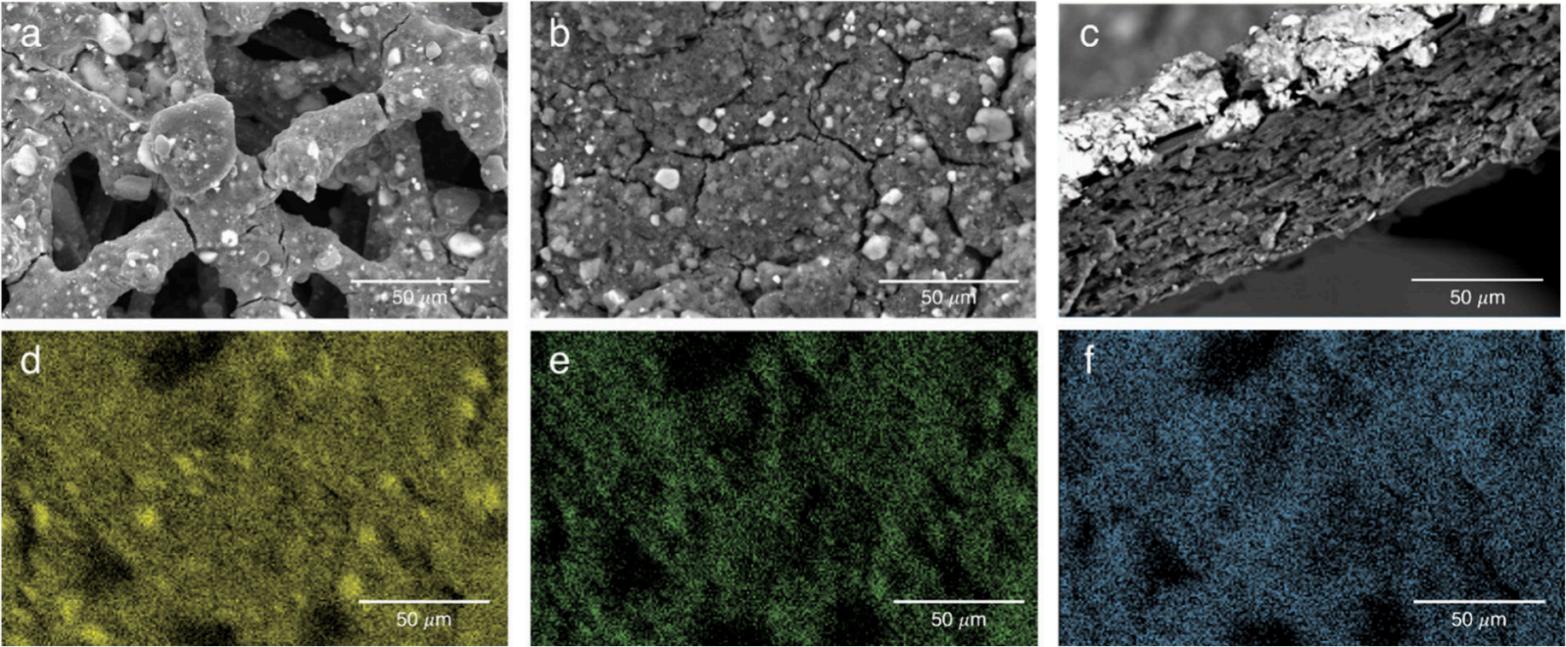
SEM images of CCS and CCM cathodes: (a) plane-view image of CCS electrode, (b) plane-view image of CCM electrode, (c) cross-section of CCM electrode, and (d–f) Pt, Ni, and Br mapping of (b). The inset scale bar is 50 μm.

The elemental mapping of the CCM electrode surface with bromide as the counter ion to the quaternary ammonium cation (i.e., before ion exchange with hydroxide) is shown in Figure 2d–f. Figure 2d,e shows the maps of the platinum and nickel particles for the Pt_3_Ni catalyst, respectively. Figure 2f is the elemental map for bromide, the ionomer counter ion. The bromide is uniformly distributed across the electrode surface, consistent with the ionomer distribution in the solvent cast method. The enhanced performance observed for the CCM cathode (Figure 1) can be attributed to its thinner, denser, and more uniform distribution of the catalyst layer, which enables better electronic and ionic connectivity with the AEM.

Figure 3a shows the long-term constant-current voltage profiles at 1.5 A cm^–2^ for cells with CCS and CCM cathodes. The CCM cathode cell has a lower initial voltage but a gradual increase in voltage over time (∼230 μV h^–1^), whereas the cell with the CCS cathode maintained a stable or slightly decreasing voltage with time. Figure 3b shows the anode voltage changes with time, which is relatively flat after the initial break-in period. Figure 3c, on the other hand, shows the cathode voltage changes with time for the CCS and CCM cathode cells. The CCM cathode potential changes with time. Thus, the voltage change in the full cell, Figure 3a, is primarily due to changes at the cathode, as shown in Figure 3c. The stability of the CCS cathode is attributed to covalent bonding between the cathode ionomer and PTL, providing better mechanical reinforcement than that in the CCM cathode, where hydrogen gas evolution likely disrupts the CCM catalyst layer adhesion to the AEM.

**Figure 3:**
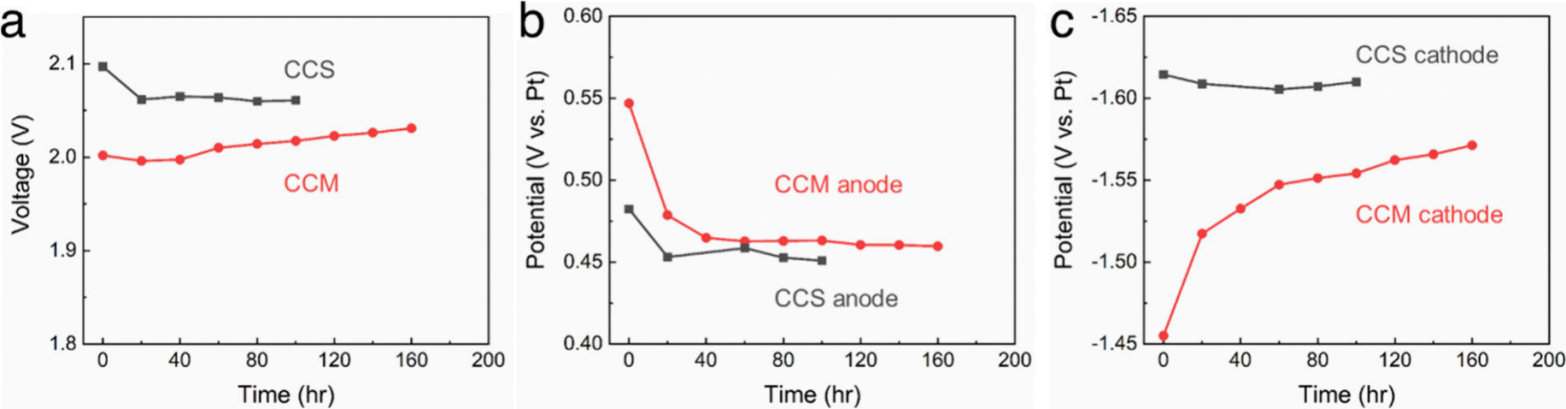
Electrolysis durability profiles with CCS and CCM cathodes measured at 1.5 A cm^–2^ and 60 °C: (a) overall cell voltage profile, (b) anode overpotential profile vs Pt pRE, and (c) cathode overpotential profile vs Pt pRE.

Electrode-specific degradation was explored using the pRE. The anode potential (Figure 3b) decreased (favorable change) during the first 40 h due to electrochemical activation of the NiFe_2_O_4_ catalyst, consistent with previous studies.^21^ The cathode potential (Figure 3c), however, steadily became more negative (unfavorable change) for CCM, suggesting degradation due to physical detachment of the catalyst layer. Physical examination confirmed the lack of adhesion of the cathode to the AEM. The limited porosity in the CCM cathode does not facilitate easy hydrogen gas escape through the cathode to the exit flow channel. The rapid buildup of hydrogen gas at the AEM/cathode interface can exert mechanical stresses within the cathode that can disrupt the catalyst layer.

The effect of hydrogen gas evolution on the adhesion of the CCM catalyst on the AEM was investigated by examining the AEM prior to and after electrolysis. Figure 4a shows a plane-view image of the electrode after deposition on the AEM. The dark colored catalyst/ionomer mixture adhered to the AEM. The electrode was then examined after electrolysis (Figure 4b). Figure 4b is an image of the electrode material on carbon paper (on the metal flow field). Figure 4c shows the AEM surface (companion picture to Figure 4a) after electrolysis showing that nearly all of the catalyst has been transferred from the AEM to the carbon paper, indicating AEM/cathode delamination. Thus, although the initial performance of the CCM cathode was better than the CCS cathode, the durability suffered due to its density and lack of chemical bonding to the AEM.

**Figure 4:**
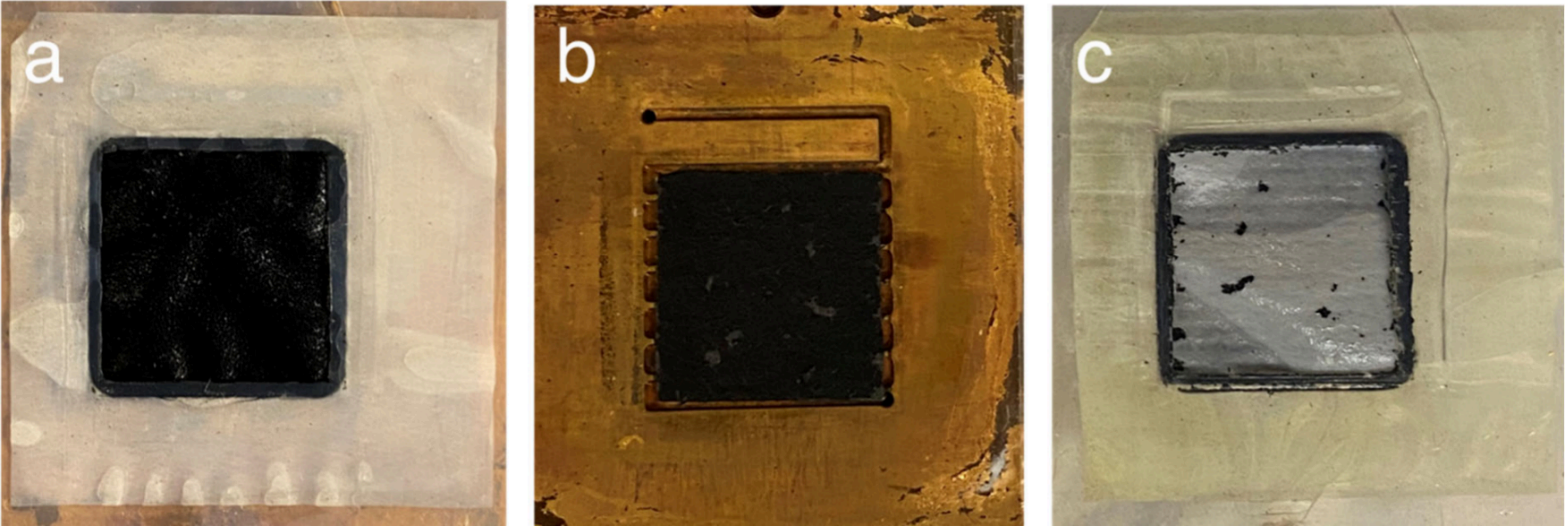
CCM cathode integrity before and after electrolysis durability test at 1.5 A cm^–2^: (a) before electrolysis and (b) after electrolysis. (b) The carbon paper with catalyst layer transferred onto it after electrolysis. (c) The AEM surface nearly void of catalyst. The active area in (b) is 2 × 2 cm^2^, slightly smaller than catalyst layer area, 2.1 × 2.1 cm^2^.

To mitigate CCM cathode degradation, TMHD cross-linker was added to the catalyst ink so as to better stabilize the cathode catalyst. The percent cross-linker in the cathode, with respect to the available sites for ionomer quaternization, varied between 0% and 5%. It is noted that increasing the cross-linking density did not increase porosity in the catalyst layer. Figure 5a shows the electrolysis voltage improved (i.e., decreased) when the cross-linking increased from 0% to 2%. However, when the cross-linking was further increased up to 5%, the cell voltage increased. Although higher cross-link density in the AEM suppresses ionomer swelling, it also results in lower conductivity and lower water uptake, both of which hinder ion and water transport within the cathode. Figure 5b shows the electrolysis voltage at 1.5 A cm^–2^ (taken from Figure 5a), showing that 2% cross-linking of the ionomer within the cathode had the most favorable cell voltage. Figure 5c shows the anode voltage (top curves) and cathode voltage (bottom curves) for the platinum reference electrode. This shows that all the performance changes between the CCM cells were due to cathode polarization effects. Figure 5d shows the EIS results for the five cells. The 2% cross-linked cathode showed the lowest ohmic and interfacial resistances (HFR reduced to 0.179 Ω cm^2^ vs 0.202 Ω cm^2^ without cross-linking). At higher cross-linking (>3%), the ionic conductivity decreased due to reduced water uptake, offsetting gains from improved gas transport.

**Figure 5:**
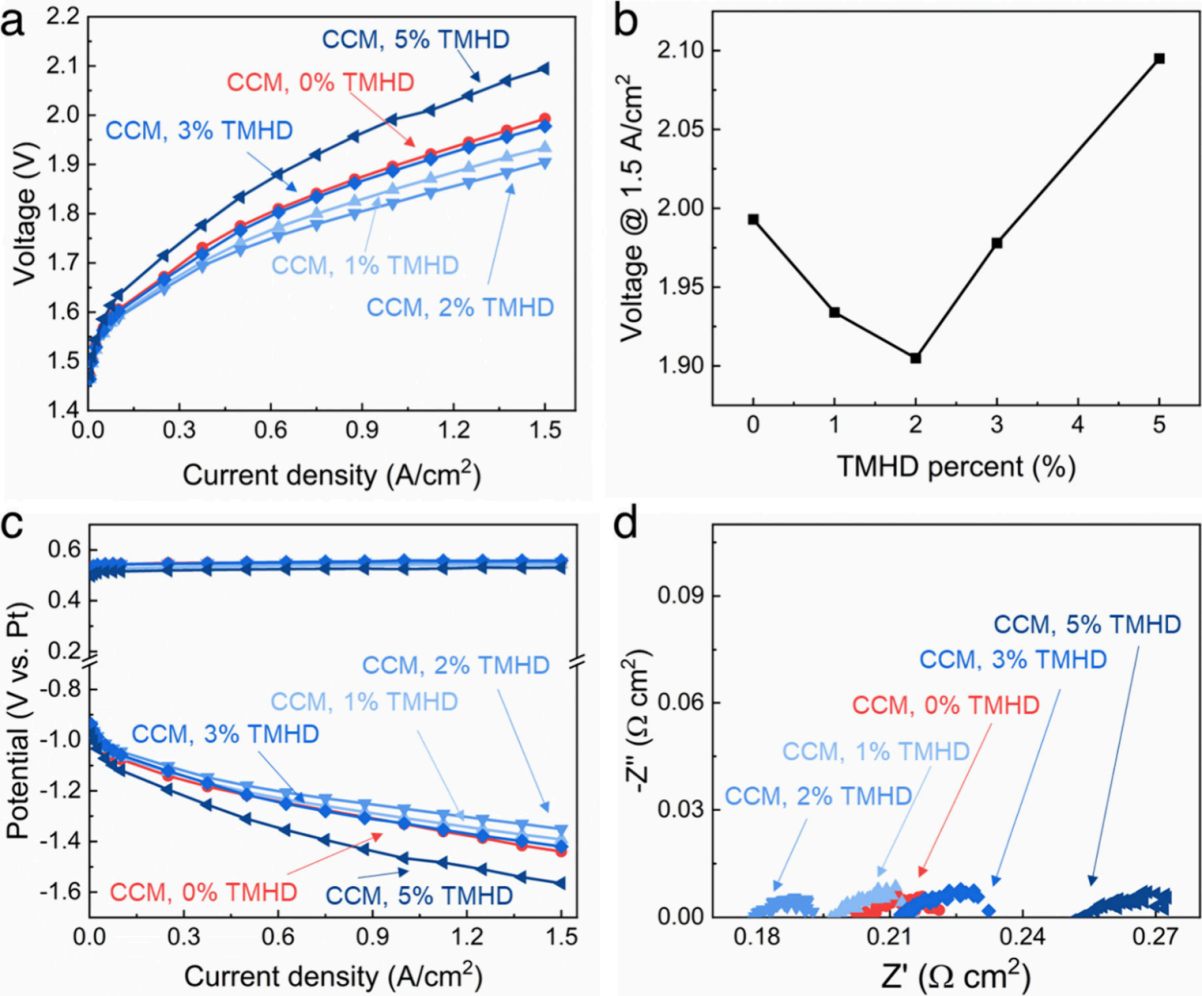
Electrolysis performance of cells with CCM cathodes with different amounts of cross-linker in the catalyst layer. Performance was measured at 60 °C with 0.1 M KOH: (a) overall cell polarization curve, (b) cell voltage recorded at 1.5 A cm^–2^ with different cross-link density within the cathode ionomer, (c) anode (top) and cathode (bottom) polarization curves measured with a Pt pRE, and (d) EIS obtained at 1.5 A cm^–2^.

The electrolysis voltage for cells with the cross-linked cathodes was investigated as a function of time. Figure 6 shows that the degradation rate decreased from 230 μV h^–1^ (0% cross-linking) to near zero (5% cross-linking). The voltage for the 5% cross-linked cathode was slightly higher than the others due to lower ionic conductivity of the cross-linked cathode ionomer; however, it showed improved catalyst layer stability with time. Anode overpotentials remained constant with time (Figure 6b), while cathode overpotentials (Figure 6c) changed with time for the cathodes with different cross-linking. The 5% cross-linked cathode, Figure 6c, achieved near steady-state cathode electrolysis voltage. Catalyst integrity of the 5% cross-linked cathode was investigated after the durability test. The CCM cathode with 0% TMHD showed that most of the catalyst particles were transferred to PTL (i.e., not adherent to the AEM), as shown in Figure 4c. The CCM with 5% cross-linking showed more than 50% of catalyst particles remained on the membrane surface (i.e., had some adhesion to the AEM). This indicates that increasing the cross-link density within the catalyst layer provides improved mechanical stability, leading to extended electrolysis durability. The increased cross-link density could also improve the hydrogen transport because the ionomer did not swell as much.^22^

**Figure 6:**
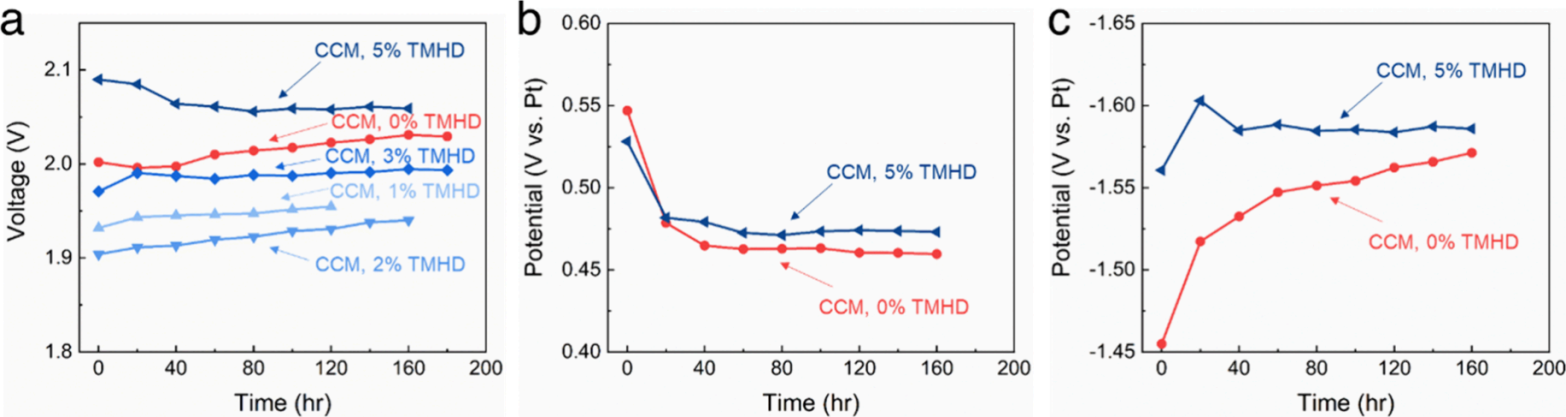
Electrolysis durability profiles of CCM cathodes with various cross-link density at 1.5 A cm^–2^ and 60 °C: (a) overall cell voltage, (b) anode overpotential vs Pt pRE for 0% and 5% cathode cross-linking, and (c) cathode overpotential profile vs Pt pRE for 0% and 5% cathode cross-linking.

The effect of the cathode catalyst layer thickness on electrolysis cell performance was investigated. Increasing the cathode thickness increased the total amount of Pt_3_Ni catalyst (i.e., total catalyst loading); however, the usable cathode area may not increase if the additional cathode material has poor ionic and water contact to the AEM (via the cathode ionomer) or the hydrogen gas produced cannot escape through the dense cathode structure. Cathodes with 0.6 and 1.2 mg cm^–2^ Pt_3_Ni loading were prepared, and the results are shown in Figure 7. Only the catalyst loading was increased, and the catalyst/ionomer ratio was held constant. SEM examination of the 0.6 mg cm^–2^ and 1.2 mg cm^–2^ cathodes show that the observable porosity and cracks in the two layers appear to be the same. This gives the thinner electrode a more favorable hydrogen escape route from the AEM/electrode interface compared with the thicker electrode. Figure 7 shows that the cathode with lower catalyst loading had better cell performance in both the low and high current density regions (Figure 7a), which could be due to a lower overall electrode resistance (i.e., electrode was thinner).^23,24^ The EIS analysis (Figure 7b) shows a decrease in both *R*_int_ and HFR with the lower catalyst loading. At 1.5 A cm^–2^, the overall cell voltage difference was 41 mV with 11 mV attributed to ohmic resistance and 30 mV attributed to interfacial effects. However, Figure 7c shows no significant difference in long-term durability between the two loading levels. The slope of the voltage vs time curves was nearly the same. These results suggest that, while catalyst loading impacts the short-term performance via improved charge transfer and reduced diffusion lengths, the long-term stability is governed more by the structural integrity and intrinsic mass transport properties than catalyst thickness alone.

**Figure 7:**
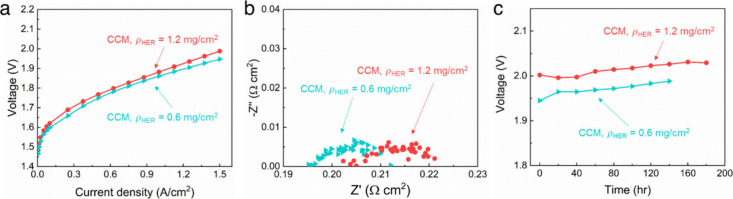
Electrolysis performance and durability of cells with CCM cathodes with different catalyst loadings. Performance was measured at 60 °C with 0.1 M KOH: (a) overall cell polarization curve, (b) EIS obtained at 1.5 A cm^–2^, and (c) electrolysis durability profile at 1.5 A cm^–2^.

In summary, the CCM cathode has potential advantages over the CCS cathodes. The AEM electrolysis cathode consumes water which is supplied by diffusion from the liquid anolyte through the AEM. Covalent bonding of the CCM cathode directly to the AEM could provide excellent mechanical integrity for the cathode and higher water and ion transport between the cathode and AEM. However, the bonding layer must balance mechanical strength with high water and ion transport. It is known that highly cross-linked anion conducting polymers have lower water content and ionic conductivity. Further, the porosity and hydrogen permeability of the CCM cathode have to be considered because the CCM cathode forms a denser, more contiguous layer than the high surface area, porous CCS cathode. The CCM cathode porosity can be increased via numerous known methods, which will be the subject of future reports.

## Conclusions

The electrochemical performance of CCM and CCS cathodes in alkaline water electrolysis was systematically compared with the integration of pRE into the MEA. The CCM cathode exhibited superior polarization performance, as shown by the lower high-frequency resistance and interfacial impedance stemming from improved contact between the catalyst layer and the AEM. Morphological analysis showed that the CCM catalyst layer was thinner and less porous than that of the CCS cathode. Durability testing at 1.5 A cm^–2^ showed that the electrolysis voltage increased over time for the CCM cathode, indicating continuous degradation. Post-electrolysis surface analysis suggested that the degradation was due to the loss of interfacial contact between the catalyst layer and the AEM. In contrast, the CCS cathode demonstrated better durability, likely due to covalent bonding between the catalyst layer and the carbon paper current collector, which was absent in the CCM configuration. The impact of incorporating an ionomer cross-linker into the cathode and varying its catalyst loading density was also explored. Cross-linking within the cathode catalyst layer with TMHD lowered the ohmic and interfacial overpotentials. However, ionomer cross-link density above 3% led to lower water uptake and ionic conductivity and increased ohmic losses. TMHD improved the long-term durability of the CCM cathodes by improving mass transport and ionomer swelling. Lowering the cathode catalyst loading improved low and high current density performances; however, the electrolysis durability did not change compared to one with higher catalyst loading. This study shows that AEM water electrolysis cell voltage can be lowered by the use of CCM cathode; however, intrinsic mass transport properties in the CCM cathode catalyst layer need to be improved for durable, energy-efficient AEM alkaline water electrolysis.
